# Loosening of the Teeth

**Published:** 1870-06

**Authors:** Leon F. Harvey


### ARTICLE IX.

### *Loosening of the Teeth.*

BY LEON F. HARVEY, A. M., M. D.

READ BEFORE THE BUFFALO DENTAL ASSOCIATION.

The loosing of the teeth, its causes and treatment, will claim our attention for a few minutes this evening. Very little can be said on the subject, for but comparatively little is known. We all know how sad it is to see fine, healthy teeth—healthy so far as freedom from decay is concerned—one after another, loosen, and finally, in many cases, actually drop from their sockets without the aid of the dentist, and we must stand by and see the ruin, powerless to effect a cure.

We do not often see this disease until after the patient has passed the age of forty-five. It is the exception and not the rule if it does occur in the youug. We find it in the strong and healthy as well as the weak and sickly. If we recollect right, it occurs more frequently in the male than the female, though we presume some writers and probably some present, would differ with us. With some the destruction is very rapid, whilst with others it takes years to effect the removal of all the teeth. We here cite a case related in the *Dental News Letter*, Vol. 8, page 128, showing the rapid progress of this disease:—

"*Case of Diseased Aveoli*, by FRANCIS CAMERON, M. D., Springwood, C. W.—What I conceive to be a rather rare case of disease and one interesting to the profession, occured in my practice some years ago. Daniel Young, aged about seventy years, consulted my medical preceptor for a disease (as he called it) of the roots of the teeth. The doctor finding it likely to prove tedious, committed it to my charge to manage under his advice. Our patient stated that he first experienced a soreness at the roots of one or two of his upper molar teeth, which shortly after became quite loose, and concurrently with the supervention of the dental looseness, a purulent discharge set in from the gums around them. The affected teeth were removed, a feat which was very easily performed, and a lotion of myrrh and borax applied to the affected gums. After the separation and removal of the diseased alveolar processes, the soft parts, under this application, soon healed. No sooner, however, had one place healed, than the disease broke out in another. It thus spread from tooth to tooth in both jaws, until all the teeth were lost and their sockets destroyed. After this he got well and lived for several years in comfortable health. Perhaps some of the readers of the *Chronicle* would enquire had he taken mercurials previously? I believe not, as his health was good for a long time before. The entire period the disease took to travel round his masticatories and disappear, was three weeks."

We should be inclined to think that this person when young had taken mercurials in some form. It is certainly a very interesting case, from the fact that the progress of the disease was so rapid.

The following is the account of a case which has lately come under our observation. But three teeth were affected and we trust that a cure has been made, though time may show us the contrary result:

A gentleman about the age of fifty complained of his teeth growing loose, that they were sore, as also were the gums around them. A fetid odor, due to purulent matter, was manifest. On examination, found that the gums were very much inflamed; that the inferior lateral incisor, the adjoining cuspidatus and the first molar of the same side, were quite loose. The tumefaction was very great. Treated the parts for some time with local depletion and the application of iodine and creosote. The patient being in good health no constitutional treatment was needed, and we considered the disease purely local. No good effects followed, and the extraction of the teeth was the last resort. Now, at the expiration of two or three months, the gums have assumed a healthy appearance and there is every indication that the disease has been arrested.

What are the causes of this affection will naturally be asked.

It may be hereditary. Ask patients whose teeth are becoming loose, if their parents lost their teeth in the same way, and some will say, yes; and also remark one or the other of their grand-parents were troubled in the same manner.

Syphilis is another cause, but that is not to be wondered at, for it attacks all the osseous structures of the body, and why not those of the mouth. Mercury, perhaps, administered years before the commencement of this disease, is often the cause.

A recent case of this kind occurs to us. A young man of the age of twenty-eight came to us in great trouble, for his left superior lateral incisor was getting quite loose; there being hardly any bony attachment, the gum around it being very much swollen and pus exuding from it. Used the usual treatment, but with no success; the case was too far gone for remedies, and the tooth dropped out. He had been suffering for a long time with catarrh and other affections, and his physician had him under a course of treatment. Soon the right lateral began to show signs of a desire to part company with him. This also, was treated in the same way, but he leaving the city, the treatment was discontinued. We have since learned that the physician now attending him ascribes all his troubles to mercury he took when young, and is treating him for the removal of the poison. The tooth, we are informed, is growing firmer, but we have no faith that it will be sound. Before he left here we had thought mercury might be the cause, but had no time for constitutional treatment, and his other affections claimed immediate attention from the physician.

Phosphorus, as used in the manufacture of matches, causes the loss of many teeth, but a distinct disease is observed here, having its own individual treatment. Blows on the teeth or gums may be an exciting cause. Tumors, malignant or non-malignant, will produce absorption of the alveolar process, and thus the teeth may be loosened. Mental exhaustion; a disordered stomach, are mentioned as causes.

Persons residing in malarial districts, who have taken large quantities of quinine, are victims of this disease, as are also the dwellers in low and damp places, such as the cellars of our cities.

Scurvy is often found to be the cause, but we do not meet with it as if we were practitioners in a seaport town where we would come in contact with sailors who often are troubled with scurvy. It is often found in women during pregnancy and about the cessation of the menses. Persons in whom tubercular consumption is manifest sometimes have this affection of the gums, but there may be no progress of the disease of the lungs till all the teeth are removed. It seems to act very much as an issue or seton. M. Oulet considers the disease beneficial in that the purulent discharge tends to suspend some more serious disease. Scrofula can also be found tampering with the teeth; retrocession of cutaneous eruptions, cessation of hæmorrhoidal discharges, are sometimes followed by a loosening of the teeth. Jourdain names it conjoint suppuration of the alveoli and gums. Our observation will not allow us to say in what teeth it is first observed, but the books say that the incisors are first attacked.

Dr. Harris mentions a form of this disease met with in persons of scrofulous habits, which he thinks differs essentially from the more common affection. In this case, the gums instead of being purple and swollen, are paler and harder than ordinary, and on being pressed exude a muco-purulent matter of a dingy white color. They often remain in this condition for years without appearing to suffer any loss of substance or to affect the alveolar process. This variety of disease of the gums is principally confined to persons who have very white teeth, and is much less likely to affect males than females. It rarely occurs before the age of eighteen or twenty, and though unquestionably the result of inflammation, the gums exhibit no inflammatory symptoms, but on the contrary are paler, less sensible, and possessed of less warmth than usual. It is never attended with tumefaction of the gums, and by absorption only in its advanced stages. Its effects are the most simple and innocent of any form of disease to which the gums are liable, but its cure is generally difficult.

Constitutional remedies, such as proper diet, exercise, air, and clothing; iodine, cod liver oil, should be prescribed. At the same time the edges of the gums should be touched with nitrate of silver.

We have introduced this form which can hardly be said to relate to the disease we have under consideration, as it is more a disease of the gums than a joint affection of the teeh and gums, to prove an aid sometime in the forming of a diagnosis.

The accumulation of tartar around the necks of the teeth and along the course of the roots, by its constant presence, produces irritation and consequent inflammation of the gums, and the alveolar process becoming involved, necrosis takes places and the teeth are lost. The deposit is more frequent when the stomach is disordered.

In some mouths we find the indications of inflammation, heat, pain, redness and swelling, and added to that pus exuding from the free margin of the gum. The breath is offensive, the patient complains of soreness at the roots of the teeth, the gums bleed on pressure ever so light; with extraction of the teeth the pain is intense and the hæmorrhage profuse. When this condition exists it is pretty certain that the process is nearly if not entirely absorbed and the only attachment the gum.

We have another condition which in our mind is the most dangerous, yet the condition of the mouth at the first glance would not warrant such an assumption. There will be but little or no indication of inflammation; very little, if any, pain from the teeth themselves, but seldom, exudation of pus; no bleeding of the gums upon touch, and but little on extraction of the teeth, with but slight pain; the walls of the sockets are slowly but surely absorbed, and the teeth are finally lost. With the first form, if the disease has appeared in but two or three teeth, we are more certain to arrest it if the cause is local, but with the second, one tooth after another is lost, almost without an exception.

We consider the second form the most difficult to treat, for we have nothing tangible to seize hold of. Where there is inflammation present the medical man knows what course to pursue, but if all the symptoms are obscure or if there is none, and yet there is disease present, but in a hidden form, he is baffled in his treatment. So it is in these two forms; the first shows a bold front and we can use vigorous measures to effect a cure, but we are sorry to confess the cures are very few. The second is like some of the old chronic cases in hospitals, remedies, have no effect upon them.

The indications for treatments in the first form, if the cause is local, are the removal of the cause; if it is due to the presence of tartar, every particle must be removed; if the inflammation is acute, the disease making rapid progress, the free use of cathartics, astringents and possibly the application of nitrate of silver. Depletion of the gums often is beneficial. The gums may be painted with iodine or a mixture of iodine and creosote. If the disease is of a chronic form, astringents should be used, tannin, myrrh, and a mouth cerate composed of nutgalls, blackberry root and honey, alternating them every two weeks. If the patient is in a low state of health, tonics should be prescribed.

When the cause is constitutional the treatment should be directed to the removal of the disease that may have brought on this affection. The teeth may often be kept secure for some time by using silver wire or surgeons' silk, binding them together.

Fintoll relates the following case:

"As an example of the trickery to which the public are subjected by its patronage of this class of dentists, permit me to digress a little in recounting the history of a case which I will preface with an extract from a daily publication:

"Loose teeth securely fastened whether arising from old age, neglect, the use of calomel, disease of the gums, or any other cause, etc., etc."

A lady advanced in years consulted me. Her lower front teeth had, by the progress of absorption of the gums and sockets, become very loose. She had yielded to the fascinations of some such advertisement as that above, and had had her teeth "securely fastened!" and so indeed they were. From the appearance which they presented I concluded that a mass of metallic amalgam miscalled "mineral cement," had been taken between the finger and thumb of the operator and pushed into and between and upon the loose teeth, tartar, decayed bone, diseased jaws, etc., just as a plasterer would take a dab of mortar on his trowel and stuff it pell-mell into the crevices of a clump of loose brick. The amalgam soon hardened and formed what I can only describe as one irregular, rough and misshapen mass, made up of the four loose lower incisors, and the two canines, these two last named, as is often the case, being comparatively firm, and forming props of support to the cement. The result of this plastering dentistry had been extensive inflammation of the gums and neighboring parts, internally as well as externally, and the patient was undergoing excessive pain and inconvenience. She was desirous that I should remove the fastening, but as I could not do so without removing the teeth also, and as she was not prepared to submit to this, she left me, and I have since lost sight of the case.

The cure of this disease that we have been considering is so important that we hope the time will come when we shall be able to stop the fearful ravages that are going on in the mouths of our patients. All we can do is try one remedy after another, with the hope that we may do some good.—*The Dental Advertiser.*

